# Effects of statins on functional capacity and cardiac remodeling in patients with heart failure and preserved ejection fraction: a randomized open-label pilot trial

**DOI:** 10.3389/fcvm.2026.1870027

**Published:** 2026-07-13

**Authors:** Artem Ovchinnikov, Alexandra Potekhina, Olga Svirida, Tatiana Arefieva, Tamila Martynyuk, Fail Ageev, Marijana Tadic, Evgeny Belyavskiy

**Affiliations:** 1 Laboratory of Myocardial Fibrosis and Heart Failure with Preserved Ejection Fraction, National Medical Research Center of Cardiology Named After Academician E. I. Chazov, Moscow, Russia; 2 Department of Clinical Functional Diagnostics, Russian University of Medicine of the Ministry of Health of the Russian Federation, Moscow, Russia; 3 Laboratory of Cell Immunology, National Medical Research Center of Cardiology Named After Academician E. I. Chazov, Moscow, Russia; 4 Department of Pulmonary Hypertension and Heart Disease, National Medical Research Center of Cardiology Named After Academician E. I. Chazov, Moscow, Russia; 5 Out-Patient Department, National Medical Research Center of Cardiology Named After Academician E. I. Chazov, Moscow, Russia; 6 University Heart Center Ulm, University Ulm, Ulm, Germany; 7 Deutsches Herzzentrum der Charité, Medizinisches Versorgungszentrum, Berlin, Germany

**Keywords:** diastolic dysfunction, diastolic stress test, heart failure with preserved ejection fraction, left ventricle, statins

## Abstract

**Background:**

Systemic inflammation is an essential pathophysiological factor of heart failure with preserved ejection fraction (HFpEF) that supports the use of statins. The purpose of the study was to determine the effects of statin therapy with atorvastatin or rosuvastatin on functional capacity, cardiac remodeling, profibrotic and inflammatory biomarkers in patients with HFpEF. Since lipophilic and hydrophilic statins may exert different effects on HF, we also compared the effects of lipophilic atorvastatin vs. hydrophilic rosuvastatin.

**Methods:**

We enrolled 59 patients with HFpEF who had no history of statin use or had not received statins for 6 months. Participants were randomly assigned to receive atorvastatin (20–80 mg a day, *n* = 30) or rosuvastatin (10–40 mg a day, *n* = 29) for 6 months. Echocardiography, 6 min walk distance (6MWTD), biomarkers of inflammation (hsCRP and MCP-1), and extracellular matrix homeostasis (PICP and sST2) were analyzed at baseline and after 6 months.

**Results:**

After 6 months of therapy, atorvastatin was associated with a greater increase in 6MWTD than rosuvastatin, but both statin groups showed comparable improvements in NYHA functional class and bicycle exercise duration. There were comparable decreases in E/e′ ratio and left atrial (LA) volume index, as well as comparable improvements in diastolic (e′ velocity enhancement on exercise) and chronotropic (heart rate enhancement on exercise) reserves in both statin groups. Atorvastatin was superior to rosuvastatin in modifying LA reserve (reservoir strain enhancement on exercise). A significant decrease in blood levels of all biomarkers was observed in the atorvastatin group but not in the rosuvastatin group. Rosuvastatin was associated with a reduction in hsCRP and MCP-1 only in subgroups of patients with baseline values above the median.

**Conclusions:**

In HFpEF, statin therapy appears to be associated with improved functional capacity, possibly by reducing LV filling pressure and favoring cardiac remodeling. Atorvastatin may provide some additional benefits compared with rosuvastatin.

**Clinical Trial Registration:**

https://rosrid.ru, identifier AAAA-A18-118022290061-2.

## 1 Introduction

Heart failure with preserved ejection fraction (HFpEF) makes up over 50% of heart failure cases. It brings significant morbidity and mortality. However, treatment options are limited due to the varied phenotypes of HFpEF (1). Most drugs do not improve these patients' exercise capacity or quality of life (2). Thus, effective therapies for HFpEF remain a major unmet medical need.

As it is more prevalent in the elderly, HFpEF shares the risk factors for atherosclerosis, and at the onset of HF, most patients have indications for statins. There is growing evidence that coronary microvascular inflammation may be a key factor in the development of HFpEF, supporting the use of 3-hydroxy-3-methylglutaryl coenzyme A (HMG-CoA) reductase inhibitors (also known as statins) in the treatment of HFpEF (3). In addition to their hypolipidemic effects, statins have been shown to stimulate nitric oxide production in endothelial cells, thereby improving nitric oxide-mediated vascular function (4, 5). Furthermore, statins have been demonstrated to exhibit anti-inflammatory (6), antihypertrophic, antifibrotic (7, 8) and antioxidant (9) properties, collectively referred to as pleiotropic effects. These properties may be advantageous in patients with HFpEF (3, 10).

According to biopsy studies, patients with HFpEF who received statins had higher protein kinase G activity and lower levels of cardiomyocyte hypertrophy, oxidative processes, and resting tension (3). An observational study found that statin-treated patients with HFpEF were less likely to develop atrial fibrillation (11). Statins have been demonstrated to improve diastolic function in patients diagnosed with coronary artery disease (CAD) (12), hyperlipidemia (13), and hypertensive heart disease (14). Several studies and meta-analyses have shown that statins reduce mortality in patients with HFpEF (15–20). However, the clinical or hemodynamic effects of statins in HFpEF have not yet been evaluated in prospective studies. Thus, this randomized study aimed to investigate the effects of statin therapy with atorvastatin and rosuvastatin on exercise capacity, cardiac remodeling, and blood levels of biomarkers of extracellular matrix homeostasis and inflammation in patients with HFpEF who were either statin-naïve or non-statin users. In addition, as recent evidence suggests that the effects of lipophilic and hydrophilic statins on heart failure (HF) may differ (21), we also compared the clinical and hemodynamic effects of the lipophilic statin atorvastatin with those of the hydrophilic statin rosuvastatin.

## 2 Materials and methods

### 2.1 Study population

This randomized, open-label, parallel-group, single-center study was conducted at the Outpatient Department of the National Medical Research Center for Cardiology in Moscow, Russia. Ambulatory participants aged 40 or older with stable HF and New York Heart Association (NYHA) class II–III, preserved LV ejection fraction (≥50%), and elevated LV filling pressure as determined by echocardiography were recruited. Eligible participants had never used statins or had discontinued them at least six months before enrollment. The main reasons for non-prescription or discontinuation were poor adherence and underuse.

Exclusion criteria included evidence of myocardial ischemia during stress echocardiography; inability to complete exercise; inadequate acoustic windows; ongoing statin therapy; chronic atrial flutter/fibrillation; ventricular paced rhythm; left bundle branch block; significant left-sided structural valve disease; significant mitral annular calcification; hypertrophic cardiomyopathy; infiltrative or inflammatory myocardial diseases; pericardial disease; and noncardiac conditions that preclude participation. The present study was approved by the Ethics Committee of the Institute of Clinical Cardiology and conducted in accordance with the Declaration of Helsinki. All patients provided written informed consent. The study was registered in the Russian National Information System of Research, Development and Technology Data of Civilian Usage (registration number АААА-А18-118022290061-2).

**Figure 1:**
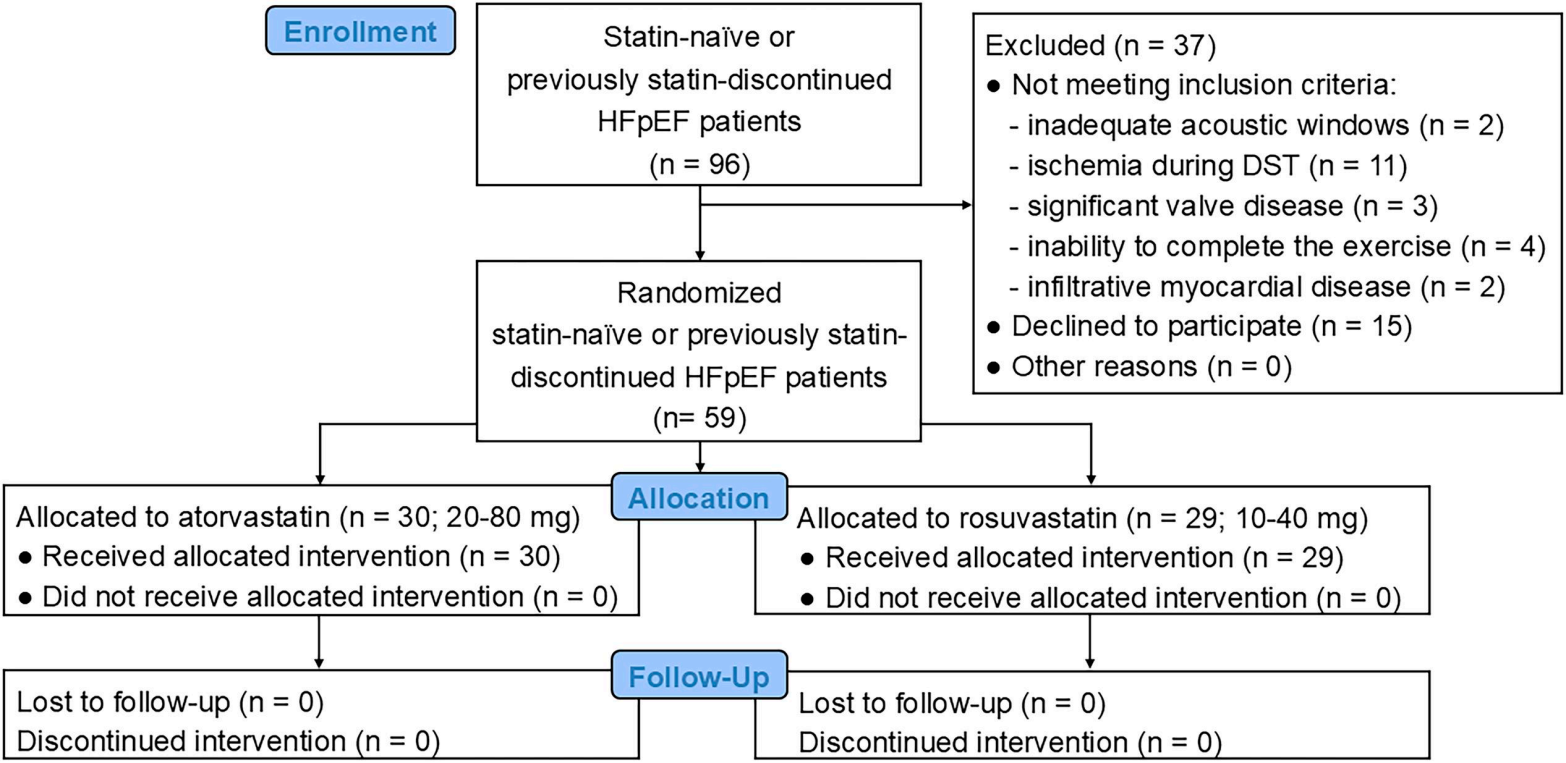
Flow chart of patient enrolment. DST, diastolic stress test; HFpEF, heart failure with preserved ejection fraction.

A total of 96 truthy statin-naïve or previously statin-discontinued subjects with HFpEF were screened between June 2017 and July 2020. Of these, 22 did not meet the inclusion criteria, and 15 declined to participate in the study's active phase. Thus, 59 participants met all criteria and were selected for the final cohort. Among them, 28 (48%) were truly statin-naïve, and 31 (52%) had stopped statin therapy for at least six months before enrollment (these are the “previously statin-discontinued subjects,” Figure 1). All HFpEF patients were at high or very high cardiovascular risk and required statin therapy (Table 1) (22).

**Table 1:** Baseline characteristics of patients with HFpEF.

Variables	Total statin group (*n* = 59)	Atorvastatin (*n* = 30)	Rosuvastatin (*n* = 29)	*P* value vs. atorvastatin
Clinical variables				
Age, *y*	67 ± 7	66 ± 9	68 ± 7	0.33
Men, *n* (%)	22 (37)	12 (40)	10 (35)	0.66
NYHA II/III, *n* (%)	45/14 (76/24)	22/8 (73/27)	23/6 (79/21)	0.59
Overweight/obesity,^a^ *n* (%)	54 (92)	28 (93)	26 (90)	0.62
Hypertension,^b^ *n* (%)	59 (100)	30 (100)	29 (100)	1.0
Paroxysmal atrial fibrillation, *n* (%)	18 (31)	8 (27)	10 (35)	0.52
Ischemic heart disease, *n* (%)	15 (25)	9 (30)	6 (21)	0.42
Previous myocardial infarction, *n* (%)	9 (15)	6 (20)	3 (10)	0.31
Myocardial revascularization, *n* (%)	14 (24)	8 (27)	6 (21)	0.59
Diabetes mellitus, *n* (%)	18 (31)	7 (23)	11 (38)	0.23
Chronic kidney disease,^c^ *n* (%)	18 (31)	9 (30)	9 (31)	0.93
Carotid atherosclerosis, *n* (%)	12 (20)	7 (23)	5 (17)	0.57
SCORE cardiovascular risk	9 (5.3–12)	9 (5–11)	9 (6.5–13.5)	0.41
NT-proBNP, pg/mL	208 (125–291)	241 (131–298)	183 (114–263)	0.29
Baseline treatments				
ACEI/ARB, *n* (%)	59 (100)	30 (100)	29 (100)	1.0
*β*-Blockers, *n* (%)	49 (83)	26 (87)	23 (79)	0.46
Diuretics, *n* (%)	46 (78)	22 (73)	24 (83)	0.39
Loop diuretics, *n* (%)	34 (58)	16 (53)	18 (62)	0.50
Thiazide/thiazide-like diuretics, *n* (%)	12 (20)	6 (20)	6 (21)	0.95
Spironolactone, *n* (%)	13 (22)	7 (23)	3 (10)	0.19
SGLT2 inhibitors	8 (14)	3 (10)	5 (17)	0.42
Statin-naïve patients, *n* (%)	28 (48)	13 (43)	15 (52)	0.52
Previously statin-discontinued patients, *n* (%)	31 (52)	17 (57)	14 (48)	0.52
Baseline echocardiographic measures				
LV ejection fraction, %	60 ± 7	60 ± 8	61 ± 6	0.15
Reduced GLS,^d^ *n* (%)	13 (22)	9 (30)	4 (14)	0.14
LV hypertrophy,^e^ *n* (%)	32 (54)	19 (63)	13 (45)	0.16
LV DD grade II-III, *n* (%)	23 (39)	12 (40)	11 (38)	0.87
Mitral E/e′	13.1 ± 3.1	13.1 ± 3.0	13.1 ± 3.2	0.99
LA dilatation,^f^ *n* (%)	43 (73)	21 (70)	22 (76)	0.62
LA dysfunction,^g^ *n* (%)	32 (54)	19 (63)	13 (45)	0.16
PASP, mm Hg	31 ± 8	32 ± 7	30 ± 8	0.36
Pulmonary hypertension,^h^ *n* (%)	21 (36)	12 (40)	9 (31)	0.48

Data are presented as the means ± standard deviations for continuous normally distributed variables, medians (25th–75th percentiles) for nonnormally distributed continuous variables, and frequencies (%) for categorical variables.

a Body mass index ≥ 25 kg/m^2.^

b Blood pressure ≥ 140/90 Hg mm.

c eGFR < 60 mL/min/1.73 m^2^.

d GLS < 16%.

e LV mass index > 115 g/m^2^ in men and >95 g/m^2^ in women.

f LA volume index ≥ 34 mL/m^2^.

g LASr < 23%.

h PAS*P* > 35 mm Hg.

ACEI, angiotensin-converting enzyme inhibitor; ARB angiotensin receptor blocker; DD, diastolic dysfunction; Е, early inflow velocity; e′, averaged annulus relaxation velocity; eGFR, estimated glomerular filtration rate; GLS, global longitudinal strain; HFpEF, heart failure with preserved ejection fraction; LA, left atrial; LASr, left atrial strain during reservoir phase; LV, left ventricular; NT-proBNP, N-terminal pro–brain natriuretic peptide; NYHA, New York Heart Association; PASP, pulmonary artery systolic pressure; RV, right ventricular; SGLT2, sodium-glucose cotransporter-2.

Eligible subjects were randomly assigned 1:1 to atorvastatin (20–80 mg daily, *n* = 30) or rosuvastatin (10–40 mg daily, *n* = 29) using an automated web-based system. Treatment allocation was concealed by sequentially numbered, opaque, sealed envelopes, opened by an external colleague after informed consent. Consistent with the principles of evidence-based therapy for patients at high risk of cardiovascular events, a nonstatin control group was not planned in the study design. Patients received statins for 6 months with monthly adherence monitoring. Counseling and educational interventions supported adherence at all visits, including counting the tablets in returned blister packs.

Since all study participants had a high or very high risk of cardiovascular events, most required the high-intensity statin regimen (atorvastatin 40–80 mg or rosuvastatin 20–40 mg) to achieve at least a 50% reduction in LDL-C from baseline or the LDL-C goal. In the atorvastatin group, 25 (83%) were up-titrated to high-intensity doses, and in the rosuvastatin group, 25 (86%) were up-titrated to high-intensity doses. Average maintenance doses were 43.3 ± 18.3 mg/day for atorvastatin and 22.8 ± 9.6 mg/day for rosuvastatin. Basic medical therapy was stable for ≥3 months before randomization and during follow-up, except for a diuretic dose increase if dyspnea worsened.

Echocardiography (at rest and during exercise), the six-minute walk test (6MWTD), and blood biomarker analyses were performed at baseline and six months after randomization. To minimize potential measurement bias, investigators performing key methods such as 6MWTD, echocardiography/diastolic stress test, and blood tests did not have access to patient allocation.

### 2.2 Echocardiography

An echocardiographic assessment was performed using a Vivid E95 ultrasound system (GE Healthcare, Horton, Norway). Ventricular dimensions, wall thickness, chamber volumes, and LV ejection fraction were determined according to current guidelines (23).

LV diastolic function was assessed by measuring the mitral inflow velocities (E, A), the averaged mitral annulus relaxation velocity (e′), the mitral E/e′ ratio, the left atrial (LA) volume index, and the tricuspid regurgitation velocity (TRV) (24). The severity of LV diastolic dysfunction (DD) was determined according to the 2016 ASE criteria for the grading of LV DD (24). Elevated LV filling pressure at rest was verified if grade II-III DD was revealed (24).

Right heart assessment included the following: right ventricle (RV) size (proximal RV outflow tract and RV basal diameters); systolic function [M-mode tricuspid annular plane systolic excursion (TAPSE)]; diastolic function [pulsed Doppler of tricuspid inflow, tissue Doppler of lateral tricuspid annulus [e′ and E/e′ ratio], and 2-dimensional measurements of right atrial [RA] volume]; pulmonary artery systolic pressure (PASP), estimated based on inferior vena cava (IVC) size and collapse (23).

LV global longitudinal strain (LV GLS) and LA global longitudinal strain (LA GLS) were performed offline. We used two-dimensional speckle-tracking echocardiography and dedicated software (Echo-Pac version 203, GE Healthcare) at 50–80 frames per second. Ventricular end-diastole was used as the baseline for LA strain curves. LA strain was calculated as the average of six LA segments in a non-foreshortened apical four-chamber view to obtain reservoir strain (LASr) (25). GLS was measured as the average systolic strain from all LV segments in apical 4-chamber, apical 2-chamber, and apical long-axis views.

All echocardiographic measures are averages of at least 3 beats.

### 2.3 Diastolic stress test (DST)

Patients performed supine cycle ergometry at 60 rpm. The exercise began with 3 min at a low workload of 25 W, followed by 3 min stages at 25 W increments to the maximal tolerated level or until limiting symptoms developed. During the test, changes in LV filling pressure (the mitral E/e′ ratio and TRV) were analyzed, as well as LV systolic (GLS), diastolic (mitral e′ velocity), and LA (LASr) functions at peak exercise compared with rest. The average of multiple beats (a minimum of 5 consecutive heart cycles) was used to minimize measurement error caused by respiratory variation during exercise. An elevated LV filling pressure during exercise was confirmed when exercise-induced increases in E/e′ ratio (an average E/e′ greater than 14) and TRV greater than 2.8 m/s were observed during DST (24).

### 2.4 Biomarkers

The blood levels of biomarkers indicative of myocardial stress [N-terminal pro-brain natriuretic peptide (NT-proBNP)], inflammation [high-sensitivity C-reactive protein (hsCRP) and monocyte chemoattractant protein-1 (MCP-1)], and extracellular matrix homeostasis [C-terminal propeptide of procollagen type I (PICP) and soluble interleukin-1 receptor-like 1 (sST2)] were analyzed at baseline and 6 months after randomization.

Biomarkers of inflammation and fibrosis were assayed in serum, while NT-proBNP was assayed in plasma. Blood samples were collected through venous puncture after a 20 min supine resting period. Samples were collected into tubes without anticoagulant to obtain serum or into tubes containing citrate anticoagulant to obtain plasma. The samples were immediately centrifuged and stored below −80 °C. NT-proBNP levels were measured using an automated electrochemiluminescence immunoassay (Roche Diagnostics, Germany). We measured hsCRP concentration by laser microscale nephelometry using a BN ProSpec laser light-scattering system (Behring, Germany). Enzyme-linked immunosorbent assay (ELISA) kits for sST2 (Critical Diagnostics, USA), MCP-1 (eBioscience, USA), and PICP (Cloud-Clone Corp., USA) were used according to the manufacturers' instructions, yielding intra- and inter-assay coefficients of variation <10%.

### 2.5 Study endpoints

The primary endpoint of the study was the change in 6MWTD after 6 months of statin treatment. Secondary objectives included changes in exercise duration during cycle ergometry, the LA volume index, the mitral E/e′ ratio at rest and during exercise, and blood biomarkers from baseline to 6 months.

### 2.6 Statistical analysis

The alteration in 6MWTD was employed to calculate the required sample size to attain adequate statistical power for the present study. According to our preliminary findings, we considered an average difference of at least 25 m between two statins to be meaningful. In accordance with the findings of our preceding study, we had anticipated the standard deviation of the differences to be 33 m (26). Given an alpha level of 0.05 (two-sided), a sample size of 29 patients per group was required to achieve 80% power.

Statistical analysis was performed using standard software (MedCalc, version 19.5.3). Data that is normally distributed is presented as the mean (standard deviation), while data that is not normally distributed is presented as the median (interquartile range). Categorical variables are shown as numbers and percentages. For normally distributed data, one-way analysis of variance was used to assess change from baseline; for nonnormally distributed data, the Wilcoxon test was used. The differences in baseline and post-treatment parameters between statin groups were tested using Student's t test for normally distributed data and the Mann–Whitney U test for nonnormally distributed data. The treatment effects are presented using point estimates and 95% confidence intervals (CIs). The correlation between continuously distributed variables was tested through univariate regression analysis. All tests were two-tailed. A value of *P* < 0.05 was considered statistically significant.

## 3 Results

### 3.1 Patient baseline characteristics and compliance

Of the 96 screened subjects with HFpEF, the study cohort included 59 patients; 30 received atorvastatin, and 29 received rosuvastatin for 6 months (Figure 1).

The mean age of the patients was 67 years, and 63% of them were women (Table 1). The subjects of the study were predominantly obese and had multiple comorbidities. A quarter of patients had coronary artery disease (CAD) with revascularization and no evidence of myocardial ischemia during the stress test. Other comorbidities included type 2 diabetes mellitus (DM) (31%), chronic kidney disease (CKD) (31%), and carotid atherosclerosis (20%). It is noteworthy that all patients with CAD and the majority of patients with DM had previously been prescribed statins but had discontinued use at least 6 months before enrollment. Twenty-three patients (39%) had grades II–III DD, which is associated with increased LV filling pressure at rest. The remaining 36 patients (61%) had grade I DD, characterized by normal LV filling pressure at rest but elevated LV filling pressure during exercise.

Of the patients, 63% were at very high risk, and 37% were at high risk for cardiovascular events. The average LDL-C level was 3.0 (2.3–3.9) mmol/L, which determined the need for statin therapy, including a high-dose regimen, among the study participants (22). Most patients had left atrial (LA) dilatation and/or dysfunction. Fifty-four percent had concentric left ventricular (LV) hypertrophy, and 22% had subtle LV systolic dysfunction despite overall preservation of EF (GLS < 16%).

The two treatment groups were comparable in demographic and hemodynamic characteristics and current medical treatment, including the proportion of statin-naive vs. previously statin-discontinued patients (Table 1). The study participants were enrolled before the introduction of modern HFpEF therapy. Consequently, only a limited number of patients were receiving sodium-glucose cotransporter 2 inhibitors, primarily due to concomitant DM (Table 1).

No patients from either group were lost to follow-up. Two patients, one from the atorvastatin group and one from the rosuvastatin group, required diuretic augmentation due to worsening dyspnea. Two patients in the atorvastatin group and one in the rosuvastatin group experienced mild muscle symptoms without elevated plasma creatine kinase during follow-up. These symptoms resolved when the statin dose was reduced. There were no differences in LDL-C changes between the atorvastatin and rosuvastatin groups, nor in liver enzyme or creatinine blood levels. No cases of clinically relevant liver enzyme elevation were reported in both statin groups. Systemic blood pressure and heart rate did not vary significantly from the baseline values in the study groups.

### 3.2 Functional capacity

**Figure 2:**
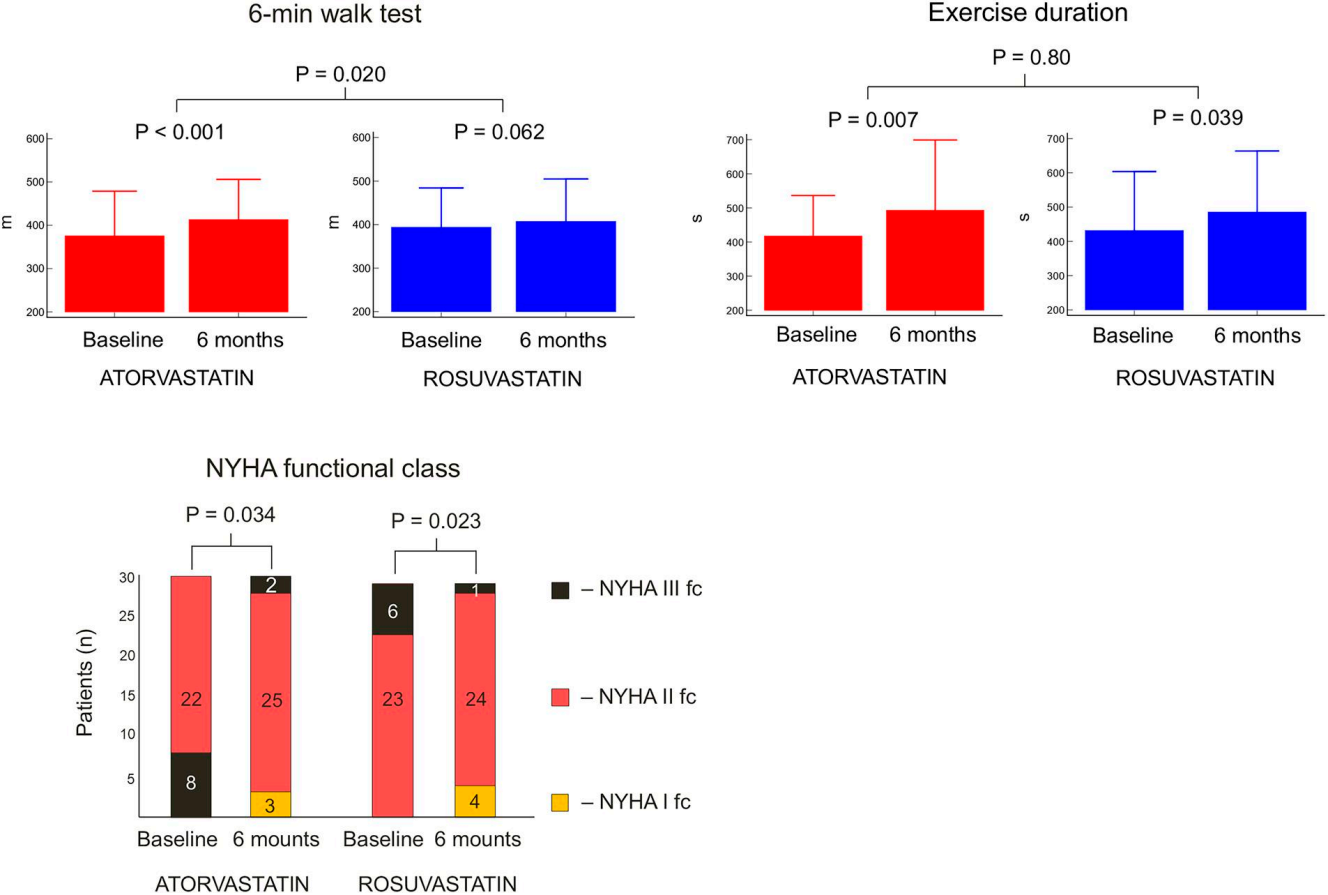
Six-minute walk distance **(A)**, bicycle exercise duration **(B)**, and NYHA functional class **(C)** at baseline and after 6 months in the atorvastatin and rosuvastatin subgroups.

After 6 months of therapy, the 6MWTD increased by 26 m (95% CI, 15 to 36 m, *P* < 0.0001) in the entire statin group (Table 2). Atorvastatin was associated with a greater improvement in 6MWTD [by 37 (95% CI, 23 to 52) m] than rosuvastatin [by 13 (95% CI, −1 to 27) m, *P* = 0.062, *P* = 0.020 for between-group differences; Figure 2A].

**Table 2:** Changes in clinical, echocardiographic and laboratory parameters and biomarker levels in the whole study group.

Variables	Baseline	Change from baseline (95% CI)	*P* value vs. baseline
Clinical parameters			
6MWTD, *m*	385 ± 96	26 (15 to 36)	<0.0001
Bicycle exercise duration, *s*	425 ± 146	65 (29 to 101)	0.0006
Biochemistry measures and biomarkers			
Total cholesterol, mmol/L	5.1 (4.2–5.9)	−1.6 (−1.8 to −1.3)	<0.0001
LDL-cholesterol, mmol/L	3.0 (2.3–3.9)	−1.4 (−1.6 to −1.2)	<0.0001
Triglycerides, mmol/L	1.6 (1.2–2.2)	−0.4 (−0.5 to −0.3)	<0.0001
Creatinine, μmol/L	78 (68–94)	2 (−1 to 5)	0.21
NT-proBNP, pg/mL	208 (125–291)	−10 (−54 to 30)	0.69
NT-proBNP > mediane, pg/mL	293 (250–423)	−104 (−176 to −25)	0.010
hsСRP, mg/L	2.0 (1.1–3.9)	−0.6 (−1.0 to −0.2)	0.008
hsСRP > mediane, mg/L	3.9 (2.8–6.7)	−1.7 (−2.5 to −0.9)	0.0004
MCP-1, pg/mL	97 (77–118)	−9 (−19 to −1)	0.039
MCP-1 > mediane, pg/mL	119 (108–158)	−27 (−39 to −15)	0.0004
PICP, ng/mL	98 (87–134)	−4 (−10 to 3)	0.27
PICP > mediane, ng/mL	134 (107–179)	−13 (−33 to 2)	0.078
sST2, ng/mL	22 (17–35)	0 (−1 to 1)	0.84
sST2 > mediane, ng/mL	35 (24–40)	−2 (−6 to −0.1)	0.038
Echocardiographic measures			
LV mass index, g/m^2^	108 ± 25	−1 (−4 to 2)	0.55
LV EDV, mL	93.3 ± 27.0	−2.6 (−6.2 to 1.1)	0.17
LV ejection fraction, %	60.3 ± 6.5	−0.4 (−2.0 to 1.2)	0.64
LV GLS, %	18.1 ± 3.3	0.1 (−0.6 to 0.7)	0.80
LAVI, mL/m^2^	38.5 ± 6.5	−2.2 (−3.2 to −1.3)	<0.0001
LASr, %	24.1 ± 6.9	−0.5 (−2.0 to 0.6)	0.31
Mitral E/e′ ratio at rest	13.1 ± 3.1	−0.9 (−1.4 to −0.3)	0.002
Mitral E/e′ ratio at peak exercise	16.2 ± 3.3	−1.4 (−2.0 to −0.8)	<0.0001
RV basal diameter, cm	4.0 ± 0.5	−0.1 (−0.2 to 0.2)	0.79
Proximal RVOT diameter, cm	3.3 ± 0.4	0.0 (−0.1 to 0.1)	0.58
RAVI, mL/m^2^	28.8 ± 7.9	−1.2 (−2.8 to 0.4)	0.14
PASP, mm Hg	31 ± 8	−6 (−8 to −4)	<0.0001
TAPSE, cm	2.1 ± 0.4	0.1 (0.05 to 0.2)	0.0013
Tricuspid Е/e′ ratio	5.5 ± 1.4	−0.1 (−0.5 to 0.3)	0.57
IVC size, cm	1.8 ± 0.4	−0.1 (−0.2 to −0.04)	0.004
IVC collapse, %	52 ± 11	4 (1 to 7)	0.007

Baseline data are presented as the mean ± standard deviation; the dynamics of variables are presented as the mean change from the baseline values (95% confidence interval). EDV, end-diastolic volume; IVC, inferior vena cava; LAVI, left atrial volume index; LDL, low-density lipoprotein; RAVI, right atrial volume index; RVOT, right ventricular outflow tract; TAPSE, tricuspid annular plane systolic excursion; 6-MWTD, 6 min walk test distance. Other abbreviations are in Table 1.

After 6 months, the duration of exercise during the bicycle test increased by 65 s (95% CI, 29 to 101 s; *P* = 0.0006) across the entire statin group (Table 2). Comparable improvements in exercise duration during the incremental bicycle test were observed in the atorvastatin and rosuvastatin groups (Figure 2B). These improvements were accompanied by a significant increase in NYHA functional class (Figure 2C). No study participants experienced a worsening of their NYHA functional class.

### 3.3 Resting echocardiographic parameters

After 6 months, a notable enhancement in echocardiographic parameters associated with LV filling pressure was observed in the entire statin group. The mean change in E/e′ ratio was −0.9 (95% CI, −1.4 to −0.3); in LA volume index, it was −2.2 (95% CI, −3.2 to −1.3 mL/m^2^); and in PASP, it was −6 (95% CI, −8 to −4) mm Hg (for all *P* < 0.01, Table 2). The atorvastatin and rosuvastatin showed comparable enhancements in these parameters: the mean change in mitral E/e′ ratio was −0.9 (95% CI, −1.8 to −0.2) in the atorvastatin group vs. −0.8 (95% CI, −1.5 to −0.1) in the rosuvastatin group (*P* = 0.80 for between-group differences); in LA volume index, it was −2.2 (95% CI, −3.2 to −1.3) mL/m2 in the atorvastatin group vs. −1.9 (95% CI, −3.2 to −0.6) mL/m2 in the rosuvastatin group (*P* = 0.48 for between-group differences); and in PASP, it was −7 (95% CI, −11 to −4) mm Hg in the atorvastatin group vs. −4 (95% CI, −8 to −2 mm Hg in the rosuvastatin group (*P* = 0.12 for between-group differences, Table 3).

**Table 3:** Changes in clinical, echocardiographic and laboratory parameters and biomarker levels in the study groups.

Variables	Atorvastatin (*n* = 30)			Rosuvastatin (*n* = 29)			
	Baseline	Change from baseline (95% CI)	*P* value vs. baseline	Baseline	Change from baseline (95% CI)	*P* Value vs. baseline	*P* value vs. Atorvastatin
Clinical parameters							
6MWTD, *m*	376 ± 103	37 (23 to 52)	<0.0001	394 ± 90	13 (−1, 28)	0.060	0.020
Bicycle exercise duration, *s*	418 ± 118	76 (23 to 129)	0.007	432 ± 172	54 (3, 105)	0.039	0.80
Maximal workload, Watts	75 (50–75)	13 (0 to 13)	0.021	75 (50–100)	0 (0 to 13)	0.11	0.68
Systolic BP, mm Hg	135 (135–145)	−3 (−10 to 5)	0.42	140 (125–145)	−5 (−10, 3)	0.15	0.80
Diastolic BP, mm Hg	85 (80–85)	−3 (−5 to 0)	0.14	85 (75–85)	−3 (−5 to 0)	0.17	0.93
Heart rate, bpm	66 ± 13	−4 (−7 to −1)	0.016	65 ± 9	−1 (−4 to 3)	0.71	0.11
Weight, kg	87 (79–106)	−1 (−2 to 0.3)	0.065	90 (78–98)	0 (−1 to 1)	0.43	0.70
Biochemistry measures							
Total cholesterol, mmol/L	5.1 (4.3–6.2)	−1.6 (−1.9 to −1.3)	<0.0001	5.1 (4.2–5.9)	−1.5 (−1.9 to −1.2)	<0.0001	0.89
LDL-cholesterol, mmol/L	3.1 (2.6–3.6)	−1.4 (−1.7 to −1.2)	<0.0001	2.9 (2.2–4.0)	−1.3 (−1.6 to −1.0)	<0.0001	0.34
Triglycerides, mmol/L	1.6 (1.1–2.2)	−0.3 (−0.5 to −0.2)	<0.0001	1.7 (1.3–2.2)	−0.4 (−0.7 to −0.3)	<0.0001	0.23
Glucose, mmol/L	5.7 (5.2–6.9)	0.0 (−0.2 to 0.3)	0.70	5.9 (5.5–6.5)	0.0 (−0.4 to 0.4)	0.82	0.89
AST, U/L	20 (16–25)	2 (−1 to 5)	0.12	20 (17–24)	2 (−1 to 6)	0.15	0.97
ALT, U/L	20 (16–25)	2 (−1 to 5)	0.084	18 (16–24)	2 (−1 to 7)	0.17	0.99
Creatinine, μmol/L	81 (68–93)	3 (−1 to 7)	0.26	75 (68–95)	1 (−4 to 4)	0.70	0.83
Echocardiographic measures							
LV mass index, g/m^2^	114 ± 28	−1 (−6, to 4)	0.75	102 ± 19	−1 (−5 to 3)	0.58	0.41
LV EDV, mL	98.6 ± 29.6	−3.9 (−9.3 to 1.5)	0.15	87.8 ± 23.2	−1.1 (−6.4 to 4.1)	0.66	0.45
LV ejection fraction, %	59.1 ± 6.7	0.6 (−2.1 to 3.2)	0.67	61.6 ± 6.2	−1.3 (−3.3 to 0.7)	0.18	0.25
LV GLS, %	17.3 ± 3.8	0.1 (−0.7 to 0.9)	0.81	18.9 ± 2.5	0.1 (−1.0 to 1.1)	0.90	0.96
LAVI, mL/m^2^	38.5 ± 7.0	−2.6 (−4.0 to −1.1)	0.001	38.6 ± 6.0	−1.9 (−3.2 to −0.6)	0.005	0.48
LASr, %	25.9 ± 7.8	−1.6 (−3.4 to 0.4)	0.098	22.4 ± 5.6	0.2 (−1.6 to 2.1)	0.81	0.17
Mitral E/e′ ratio	13.1 ± 3.0	−0.9 (−1.8 to −0.2)	0.027	13.1 ± 3.2	−0.8 (−1.5 to −0.1)	0.027	0.80
RV basal diameter, cm	4.0 ± 0.6	−0.1 (−0.2 to 0.0)	0.11	4.0 ± 0.5	0.0 (−0.1 to 0.1)	0.60	0.21
Proximal RVOT diameter, cm	3.3 ± 0.4	0.0 (−0.1 to 0.1)	0.59	3.2 ± 0.4	0.0 (−0.1 to 0.1)	0.83	0.82
RAVI, mL/m^2^	30.2 ± 8.2	−1.7 (−4.0 to 0.6)	0.14	27.3 ± 7.4	−0.6 (−2.9 to 1.7)	0.60	0.48
PASP, mm Hg	32 ± 7	−7 (−10 to −4)	0.0002	30 ± 8	−4 (−7 to −1)	0.006	0.11
TAPSE, cm	2.1 ± 0.4	0.1 (−0.01 to 0.2)	0.075	2.1 ± 0.3	0.2 (0.1 to 0.3)	0.008	0.25
Tricuspid Е/e′ ratio	5.5 ± 1.6	0.2 (−0.4 to 0.7)	0.57	5.4 ± 1.2	−0.4 (−0.9 to 0.1)	0.13	0.14
IVC size, cm	1.9 ± 0.4	−0.2 (−0.3 to −0.1)	0.004	1.6 ± 0.5	−0.1 (−0.2 to 0.1)	0.27	0.53
IVC collapse, %	51 ± 9	8 (4 to 11)	0.0002	54 ± 12	0 (−4 to 4)	0.98	0.004

Baseline data are presented as the mean ± standard deviation; the dynamics of variables are presented as the mean change from the baseline values (95% confidence interval). ALT, alanine aminotransferase; AST, aspartate aminotransferase; BP, blood pressure. Other abbreviations are in Tables 1, 2.

No changes in LV ejection fraction, volumes, mass, or speckle-tracking parameters were observed in either group.

Although only 13 out of 59 (22%) patients showed echocardiographic signs of RV dysfunction (as indicated by a TAPSE of ≤1.7 cm), treatment with statins was also associated with RV systolic function improvement, as evidenced by an increase in TAPSE (*P* = 0.001) and central venous pressure descent (an increase in IVC collapse with sniff and a decrease in IVC size, *P* < 0.01 for both parameters, Table 2). In patients with baseline RV systolic dysfunction, statin therapy was associated with a significantly greater increase in TAPSE than in those with normal baseline RV function [+17% [95% CI, 9 to 24] % vs. +3% [95% CI, −1 to 7] %, respectively; *P* = 0.004]. Furthermore, the baseline TAPSE value was inversely correlated with the changes in TAPSE during statin therapy (*r* = −0.37, *P* = 0.004).

The atorvastatin and rosuvastatin groups showed similar changes in right heart parameters. However, there was a greater increase in IVC collapse with a sniff in the atorvastatin group (*P* = 0.004 for between-group differences; see Table 3). This difference is likely due to a more pronounced decrease in central venous pressure.

### 3.4 Exercise hemodynamics (diastolic stress test)

**Figure 3:**
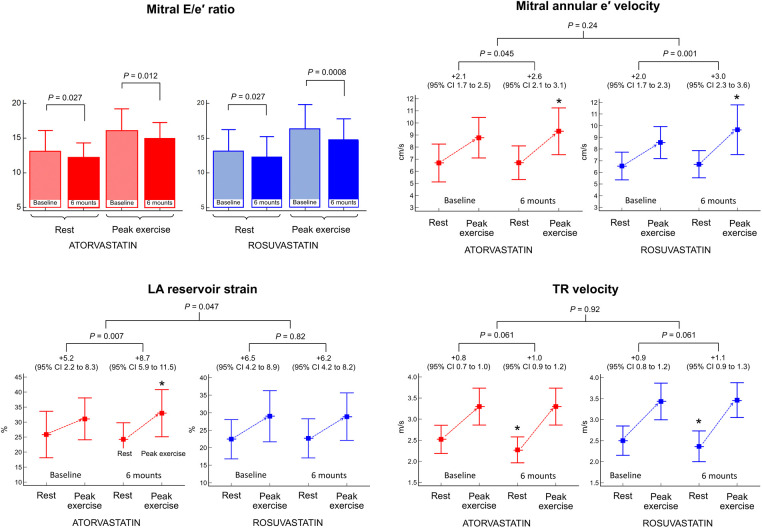
Changes in the mitral E/e′ ratio **(A)**, mitral annular e′ velocity **(B)**, left atrial (LA) reservoir strain **(C)**, and tricuspid regurgitation [TR] velocity **(D)** during cycle exercise at baseline and after 6 months. *P < 0.05 vs. baseline.

All study participants underwent a stress test, including stable patients with CAD who showed no new signs of ischemia during stress echocardiography. At baseline, exercise was associated with significant increases in both LV filling pressure (estimated using the mitral E/e′ ratio) and PASP (estimated using TRV) (Figure 3A).

**Figure 4:**
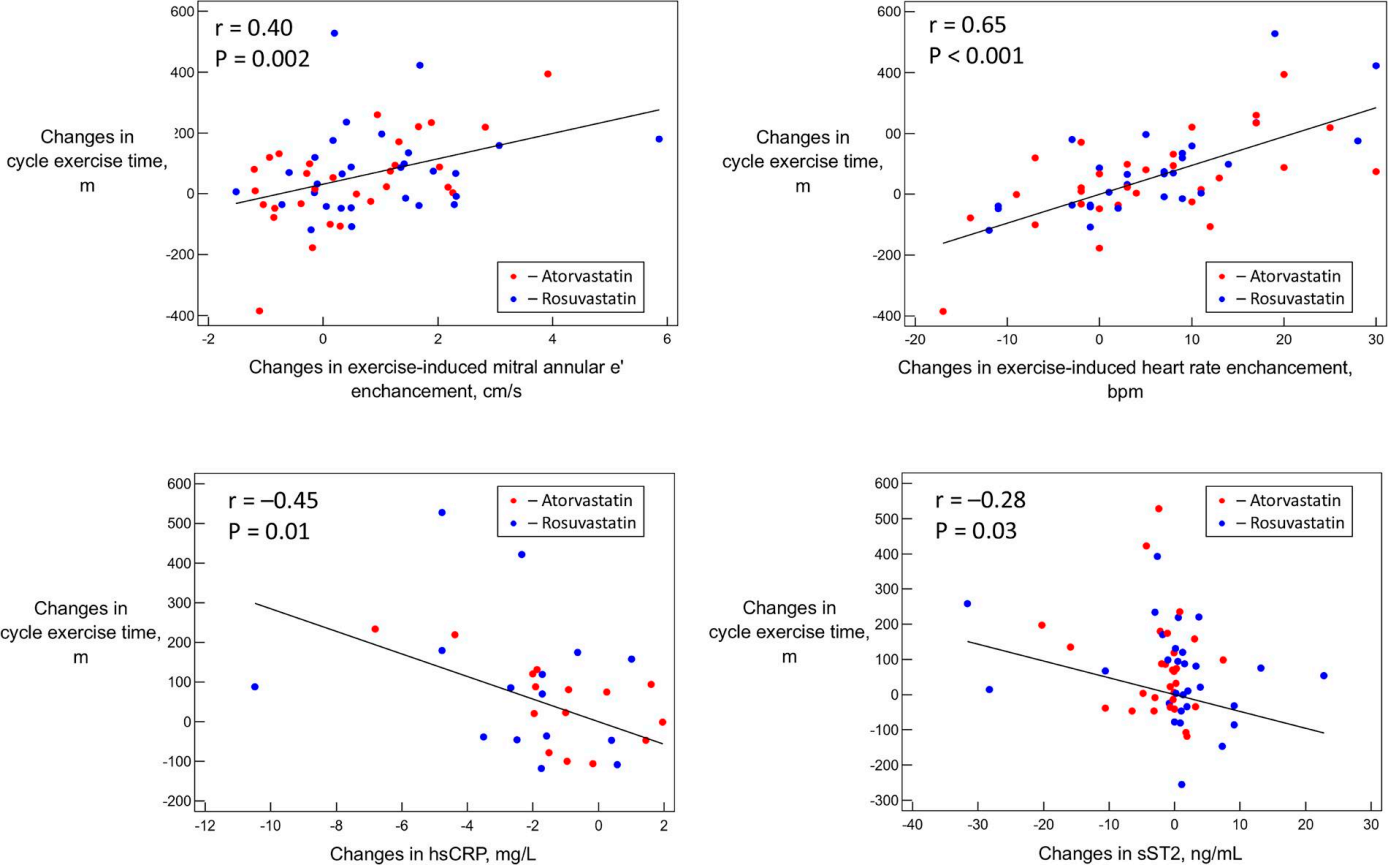
Correlations between changes in exercise duration (from baseline values to 6-month values) and exercise-induced mitral annular e′ enhancement **(A)**, exercise-induced LASr enhancement **(B)**, hsCRP blood levels (only patients with levels above the median at baseline) **(C)**, and sST2 blood levels **(D)** at 6-month diastolic stress test in the entire study cohort. e′, averaged annulus relaxation velocity; hsCRP, high sensitivity C-reactive protein; LASr, left atrial strain during reservoir phase; sST2, soluble interleukin 1 receptor-like 1.

After 6 months, the E/e′ ratio decreased significantly at rest and at the peak of exercise in the entire statin group, as well as in the atorvastatin and rosuvastatin subgroups (see Tables 2, 4 and Figure 3A). In the whole statin group, the improvement in LV filling pressures was accompanied by an increase in mitral e′ velocity enhancement during exercise (reflecting improvement in LV diastolic reserve), an increase in global longitudinal strain (GLS) enhancement during exercise (reflecting improvement in LV systolic reserve), and an increase in exercise heart rate (reflecting improvement in chronotropic reserve; see Table 4). There were no differences in the dynamics of these reserves between the atorvastatin and rosuvastatin groups (Figure 3B, Table 4). However, atorvastatin demonstrated a more marked increase in LASr enhancement during exercise (improvement in LA reserve) than rosuvastatin (*P* = 0.047; Figure 3C). The changes in exercise-induced mitral E′ velocity enhancement and heart rate enhancement (reflecting changes in LV diastolic and chronotropic reserves, respectively) were correlated with exercise duration achieved during statin therapy (Figure 4, panels A and B).

**Table 4:** Dynamics of exercise echocardiographic measures in the study groups.

Variables	Total statin group (*n* = 59)		Atorvastatin (*n* = 30)		Rosuvastatin (*n* = 29)	
	Baseline of study	6 months of study	Baseline of study	6 months of study	Baseline of study	6 months of study
HR, bpm						
Rest	66 ± 11	63 ± 8	66 ± 13	62 ± 7	65 ± 9	64 ± 9
*Δ* Rest-to-Peak^a^	34 (31 to 37)	40 (37, 43)	35 (31 to 39)	40 (36 to 45)	33 (29, 37)	39 (34 to 44)
Changes in *Δ* Rest-to-Peak		6 (3 to 8)**		5 (1 to 9)*		6 (2 to 10)**
Mitral E/e′ ratio						
Rest	13.1 ± 3.1	12.2 ± 2.5*	13.1 ± 3.0	12.2 ± 2.2*	13.1 ± 3.2	12.3 ± 2.9
*Δ* Rest-to-Peak^a^	3.1 (2.6 to 3.6)	2.6 (2.1 to 3.1)	3.0 (2.3 to 3.8)	2.8 (2.1 to 3.5)	3.2 (2.5 to 4.0)	2.4 (1.7 to 3.2)*
Changes in *Δ* Rest-to-Peak		−0.5 (−1.1 to 0.0)*		−0.2 (−0.9 to 0.5)		−0.8 (−1.8 to 0.1)
TR velocity, m/s						
Rest	2.5 ± 0.3	2.3 ± 0.3**	2.5 ± 0.3	2.3 ± 0.3**	2.5 ± 0.3	2.3 ± 0.3*
*Δ* Rest-to-Peak^a^	0.9 (0.8 to 1.0)	1.1 (0.9 to 1.2)	0.8 (0.7 to 1.0)	1.0 (0.9 to 1.2)	0.9 (0.8 to 1.1)	1.1 (0.9 to 1.3)
Changes in *Δ* Rest-to-Peak		0.2 (0.1 to 0.3)**		0.2 (0.0 to 0.4)		0.2 (0.0 to 0.3)
Mitral e′, cm/s						
Rest	6.6 ± 1.4	6.7 ± 1.3	6.7 ± 1.6	6.7 ± 1.4	6.5 ± 1.2	6.7 ± 1.2
*Δ* Rest-to-Peak^a^	2.1 (1.8 to 2.3)	2.8 (2.4 to 3.2)	2.1 (1.7 to 2.5)	2.6 (2.1 to 3.1)	2.0 (1.7 to 2.3)	3.0 (2.3 to 3.6)
Changes in *Δ* Rest-to-Peak		0.7 (0.4 to 1.1)**		0.5 (0.0 to 1.0)*		0.9 (0.4 to 1.5)**
LASr, %						
Rest	24.1 ± 6.9	23.5 ± 5.6	25.9 ± 7.8	24.3 ± 5.5	22.4 ± 5.6	22.7 ± 5.6
*Δ* Rest-to-Peak^a^	5.9 (4.0 to 7.7)	7.4 (5.7 to 9.1)	5.2 (2.2 to 8.3)	8.7 (5.9 to 11.5)	6.5 (4.2 to 8.9)	6.2 (4.2 to 8.2)
Changes in *Δ* Rest-to-Peak		1.5 (−0.4 to 3.4)		3.5 (1.1 to 5.9)**		−0.3 (−3.2 to −2.6)^§^
LV GLS, %						
Rest	18.1 ± 3.3	18.2 ± 3.4	17.3 ± 3.8	17.4 ± 3.7	18.9 ± 2.5	19.0 ± 2.9
*Δ* Rest-to-Peak^a^	3.6 (2.9 to 4.3)	4.5 (3.7 to 5.2)	3.7 (2.6 to 4.8)	4.4 (3.5 to 5.2)	3.5 (2.7 to 4.3)	4.5 (3.3 to 5.8)
Changes in *Δ* Rest-to-Peak		0.9 (0.1 to 1.6)*		0.7 (−0.3 to 1.6)		1.1 (−0.2 to 2.3)
LV EDV, mL						
Rest	93 ± 27	91 ± 24	99 ± 30	95 ± 27	88 ± 23	87 ± 19
*Δ* Rest-to-Peak^a^	12 (9 to 5)	11 (9 to 14)	13 (10 to 16)	12 (8 to 15)	11 (7 to 16)	11 (7 to 15)
Changes in *Δ* Rest-to-Peak		−1 (−4 to 2)		−1 (−5 to 3)		−1 (−6 to 5)

Baseline data at rest are presented as the mean ± standard deviation; the dynamics of variables and cardiac reserves are presented as the mean change from the baseline values (95% confidence interval).

a The changes in all parameters from rest to peak exercise in both groups, at the initial visit and after 6 months, were highly significant (Р < 0.001).

* *P* < 0.05.

** *P* < 0.01 vs. baseline (intragroup differences).

§ *P* < 0.05 vs. atorvastatin group (intergroup differences).

Abbreviations are as in Tables 1, 2.

After 6 months, the resting TRV had decreased, although the amplitude of the exercise-induced TRV elevation was greater than that in the baseline stress test in both statin groups (Table 4). Consequently, the TRV values at the peak of exercise during the six-month follow-up did not differ significantly from those at the baseline visit (Figure 3D).

No principal changes in the dynamics of GLS and LV end-diastolic volume during exercise (which correspond to LV systolic function and preload reserves, respectively) vs. baseline assessment were observed in either statin group (Table 4).

### 3.5 Biomarkers

After 6 months, a significant decrease in blood levels of inflammation biomarkers (hsCRP and MCP-1) was observed across the entire study cohort (*P* < 0.05 for both markers; Table 2).

The plasma levels of the profibrotic biomarkers PICP and sST2 remained unchanged in the entire statin group throughout the study (Table 2). However, in patients with baseline levels of these markers above the median, statin therapy was associated with a significant reduction in levels at the 6-month follow-up (*P* = 0.078 for PICP and *P* = 0.038 for sST2; Table 2).

A significant decrease in hsCRP, MCP-1, PICP, and sST2 blood levels was observed in the atorvastatin group after six months of therapy (*P* < 0.05 for all) but not in the rosuvastatin group. Furthermore, treatment with atorvastatin was associated with a significant decrease in sST2 and a trend towards a reduction in PICP compared with rosuvastatin (*P* = 0.008 and *P* = 0.067 for between-group differences, respectively; Table 5). Nevertheless, rosuvastatin was associated with a significant reduction in hsCRP and MCP-1 in subgroups of patients with hsCRP or MCP-1 levels above the median at baseline (*P* < 0.05 for both, Table 5).

**Table 5:** Dynamics of the level of biomarkers in the study groups after 6 months of therapy.

Variables	Atorvastatin (*n* = 30)			Rosuvastatin (*n* = 29)			
	Baseline^a^	Change from baseline (95% CI)	*P* Value vs. baseline	Baseline^a^	Change from baseline (95% CI)	*P* Value vs. baseline	*P* Value vs. Atorvastatin
NT-proBNP, pg/mL	241 (131–298)	−15 (−76 to 41)	0.70	183 (114–263)	−6 (−69 to 52)	0.82	0.88
NT-proBNP > mediane (>208 pg/mL)	293 (258–441)	−106 (−194 to −15)	0.023	291 (230–409)	−99 (−292 to 109)	0.20	0.21
hsСRP, mg/L	2.0 (1.1–3.7)	−0.5 (−1.0 to −0.02)	0.046	1.7 (0.9–4.3)	−0.7 (−1.7 to 0.1)	0.098	0.73
hsСRP > mediane (>2.0 mg/L)	3.7 (2.6–5.5)	−1.1 (−2.1 to −0.04)	0.045	4.3 (3.5–7.4)	−2.2 (−3.6 to −1.2)	0.0009	0.19
MCP-1, pg/mL	99 (90–115)	−15 (−31 to −2)	0.026	94 (71–129)	−6 (−17 to 11)	0.57	0.24
MCP-1 > mediane (>97 pg/mL)	115 (106–168)	−35 (−67 to −13)	0.003	130 (110–156)	−22 (−32 to −6)	0.030	0.23
PICP, ng/mL	99 (92–127)	−9 (−17 to −1)	0.039	97 (85–144)	2 (−8 to 14)	0.61	0.067
PICP > mediane (>98 ng/mL)	127 (104–167)	−17 (−49 to −6)	0.013	164 (122–200)	−2 (−41 to 23)	0.89	0.27
sST2, ng/mL	22 (17–35)	−1 (−3 to −0.1)	0.033	22 (16–27)	1 (−1 to 3)	0.14	0.008
sST2 > mediane (>22 ng/mL)	35 (26–40)	−3 (−8 to −1)	0.004	35 (24–38)	0 (−14 to 4)	0.81	0.11

a There were no significant differences in baseline levels of all biomarkers between groups. Baseline data are presented as medians (interquartile ranges), and the dynamics of the variables are presented as mean changes from baseline (95% confidence intervals). hsCRP, high sensitivity C-reactive protein; MCP-1, monocyte chemoattractant protein-1; NT-proBNP, N-terminal pro–brain natriuretic peptide; PICP, C-terminal propeptide of procollagen type I; sST2, soluble interleukin 1 receptor-like 1.

Changes in hsCRP blood levels during statin therapy were found to be directly correlated with changes in PASP, which may indicate changes in left ventricular filling pressure (*r* = 0.34, *p* = 0.009). Changes in hsCRP levels above the baseline median, as well as changes in sST2 levels, were inversely correlated with exercise duration achieved during follow-up (Figures 4C,D).

Although there was a significant reduction in LDL-C in both groups (Table 3), single-factor correlation analysis showed no significant correlation between changes in LDL-C and 6MWTD, or other key clinical and echocardiographic parameters (Additional File 1).

Plasma levels of the myocardial stress marker NT-proBNP remained unchanged in both statin groups during the study period. However, in patients with baseline NT-proBNP levels above the median, 6-month statin therapy was associated with a 36% reduction in the atorvastatin group (*P* = 0.023) and a 31% reduction in the rosuvastatin group (*P* = 0.21; Table 5).

## 4 Discussion

Here, we demonstrate that moderate-to-high-intensity statin treatment with atorvastatin or rosuvastatin in well-defined, ambulatory, chronic, stable HFpEF patients may be associated with increased functional capacity and exercise duration. These clinically beneficial effects were accompanied by a decrease in LV filling pressure and improvements in key cardiac reserves, whereas atorvastatin may offer additional benefits compared with rosuvastatin.

The novel HFpEF paradigm posits that proinflammatory comorbidities, including metabolic disorders, hypertension, DM, and renal insufficiency, act as triggers for a low-grade systemic inflammatory state. This state is characterized by myocardial infiltration with activated leukocytes and cardiac fibrosis (3). Microvascular inflammation has been shown to trigger oxidative stress and impair the nitric oxide-cyclic guanosine monophosphate-protein kinase G signaling pathway, leading to cardiomyocyte hypertrophy and altered myofilament protein phosphorylation (27). Statins have been shown to rapidly improve endothelial redox balance and restore nitric oxide bioavailability, independent of their low-density lipoprotein-lowering effect (28). Many studies have reported consistent positive effects of statins on mortality in patients with HFpEF (15–17, 29), including those without CAD (30). These results were corroborated by numerous meta-analyses, which consistently demonstrated that statin therapy was associated with reduced mortality in this population (18–20). However, to our knowledge, we are the first to demonstrate an improvement in exercise duration and functional capacity with statin therapy in a well-defined population of patients with HFpEF in a prospective study. This is particularly important since statin-associated muscle symptoms can hide the benefits of statins for elderly patients with HFpEF. Despite the poor adherence to statin therapy among patients with HFpEF, we improved adherence through counseling and education combined with frequent, comprehensive monitoring.

In the present study, administration of atorvastatin or rosuvastatin was associated with a reduction in LV filling pressure (assessed by the E/e′ ratio) at rest and during peak exercise. This reduction was accompanied by LA reverse remodeling and improvement in principal cardiac reserves, including chronotropic, LV diastolic and contractile, and LA reservoir reserves (the latter only in the atorvastatin group). An enhancement in cardiac reserves in HFpEF is of considerable significance, as HFpEF is characterized by diminished reserve capacity across multiple domains of cardiovascular function that contribute to exercise intolerance (31).

The presence of RV dysfunction in HFpEF is an independent predictor of mortality, making it a potential therapeutic target (32). Given that microvascular inflammation and endothelial dysfunction involve systemic factors, there may be simultaneous involvement of the RV, leading to RV remodeling and dysfunction. In the present study, the statin treatment was associated with improvements in pulmonary and right heart hemodynamics in HFpEF patients, mainly in the subgroup with initial pulmonary and right heart impairments. Interestingly, statin therapy led to a decrease in resting PASP but, paradoxically, to a significant increase in PASP during exercise. Given the marked reduction in the mean LA pressure (an important contributor to PA pressure) at the peak of exercise after treatment, along with improvements in RV contractility, the exercise-induced elevation of PASP probably arises from enhanced RV contractile reserve.

Given the variety of reserve limitations observed in HFpEF and statins' capacity to enhance essential cardiac reserves, we hypothesize that a unifying process underlies these reserve capacities. This process may involve nitroso-oxidative stress and/or impaired nitric oxide (NO)/cyclic guanosine monophosphate (cGMP)-dependent signaling pathways (3). The beneficial effects of statins may result from targeting this pathological process.

According to our data, therapy with atorvastatin or rosuvastatin was associated with restoration of the exercise-induced increase in e′ velocity. The change in e′ response to exercise was directly correlated with exercise duration dynamics, demonstrating improvement in diastolic reserve and its role in adequate exercise tolerance. The ability of statins to improve LV DD has been demonstrated in several cardiovascular populations, including patients with CAD (12) and hyperlipidemia (13). In an earlier observational study, we demonstrated that statin therapy prevented the progression of LV DD and the transition to HFpEF in patients with asymptomatic hypertensive heart disease (14).

The beneficial effects of statins on diastolic function may be mediated by their well-documented anti-inflammatory and antifibrotic properties. Numerous experimental studies support the improvement of LV DD with statins by interfering with the inflammatory and fibrotic cytokine network (6–8, 33–37). CRP is one of the most sensitive markers of inflammation. In patients with HFpEF, CRP is associated with an increased risk of developing HFpEF and poor outcomes (38). According to the Korean Acute Heart Failure Registry, statin therapy affects the prognosis of patients with HFpEF, depending on baseline CRP levels (39). In the present study, a direct correlation was observed between the extent of decrease in hsCRP level achieved during statin therapy and the more pronounced positive dynamics of exercise duration and PASP. This finding links the anti-inflammatory properties of statins with clinical and hemodynamic effects.

In the present study, lipophilic atorvastatin therapy consistently reduced inflammatory and profibrotic biomarkers, whereas hydrophilic rosuvastatin reduced only inflammatory biomarkers, and only in participants with higher baseline levels. In the Stat-LVDF study, lipophilic pitavastatin, but not hydrophilic rosuvastatin, delayed an increase in the LV wall stress biomarker BNP in patients with hypercholesterolemia and asymptomatic LV DD (40). Meta-analyses have shown that lipophilic statins are more effective than hydrophilic statins in decreasing inflammation (21, 41) and reducing all-cause mortality and hospitalizations due to HF exacerbation (19, 41) in patients with HF.

Hydrophilic statins, such as rosuvastatin and pravastatin, exhibit greater hepatoselectivity than lipophilic statins. In contrast, lipophilic statins undergo passive diffusion through hepatic and non-hepatic tissues, including cardiac cells, such as macrophages, cardiomyocytes, and fibroblasts (42). Therefore, lipophilic statins may have more significant pleotropic effects than hydrophilic statins because they can penetrate extrahepatic tissues, such as hypertrophic myocardium. According to the present study, lipophilic atorvastatin was superior to hydrophilic rosuvastatin in improving variables from clinical (6MWTD, which was a primary endpoint), functional/hemodynamic (LA reserve and central venous pressure), and biomarker variables.

It has been posited that lipophilic statins, due to their more pronounced pleiotropic properties, may be more toxic and lead to a greater number of adverse effects. However, this hypothesis is not supported by either the clinical trial data (43, 44) or the present study: lipophilic atorvastatin did not differ from hydrophilic rosuvastatin in the incidence of adverse effects or in changes in transaminase, glucose, and creatinine levels.

Most patients with HFpEF are at high or very high cardiovascular risk and are candidates for intensive statin therapy (22). However, lipid-lowering therapy remains challenging in treating cardiovascular diseases, including HFpEF. Statins are the most commonly underused and underdosed drug class, especially in elderly patients (45). Recent large RCTs found that 31%–47% of participants did not receive lipid-lowering medications (46). All patients in our study were candidates for statin therapy based on risk factors and LDL-C levels. However, half of the patients were statin-naïve at the start of the study, while the other half had previously stopped taking prescribed statins. Furthermore, 16% of the screened subjects refused to participate in the study due to their unwillingness to take statins. Poor adherence is the main barrier to statin therapy. It is associated with concerns about side effects and a lack of apparent treatment benefits. Other factors include cost and the complexity of the treatment (45). However, a recent Hong Kong observational retrospective study found that initiating statin therapy in older adults (ages ≥75 years) without cardiovascular disease was associated with a substantial reduction in cardiovascular events without an increased risk of major adverse events (47). Additionally, an observational study found that statin therapy in naïve patients with HFpEF and without atherosclerotic cardiovascular disease was associated with reduced all-cause mortality and heart failure hospitalization compared with statin nonusers (48).

### 4.1 Study limitations

The study's primary limitations include the absence of a placebo/nonstatin control group, the unblinded design, the relatively small number of participants, and the single-center design.

It is noteworthy that all participants exhibited a high or very high risk of cardiovascular events and required statin treatment. Given that the natural history of HFpEF presents gradual clinical and hemodynamic deterioration (14, 49), the significant improvement in functional parameters across all key domains—clinical (functional capacity), functional (E/e′ ratio and PASP), morphological (LA volume index), and biological (biomarkers)—suggests a beneficial effect of statins in HFpEF.

Since statins have been shown to improve myocardial function and prognosis in patients with CAD, the observed improvements may reflect optimized treatment of underlying ischemia rather than specific benefits in HFpEF. Nevertheless, we attempted to minimize this effect by excluding patients who experienced myocardial ischemia during the diastolic stress test. Additionally, total revascularization was achieved in all cases of CAD except one.

Although peak oxygen uptake (VO₂ peak) during the cardiopulmonary exercise test is the most objective method for assessing subjects' functional capacity, we used 6MWTD and supine cycle ergometry (as part of the diastolic stress test) to assess exercise capacity. Nevertheless, 6MWTD and supine cycle ergometry yielded consistent beneficial results in both statins’ groups, which, combined with the apparent improvement in echocardiographic parameters related to LV filling pressure, allows us to assume the ability of statins to improve functional capacity in HFpEF.

The present pilot study included an insufficient number of patients for multivariate regression analysis, which could be relevant for assessing the significance of possible confounding factors, such as changes in weight, BP, or LDL-C, in the context of other factors with respect to end points. Nevertheless, given the lack of significant weight loss and BP reduction with statin therapy, as well as the absence of significant correlations between LDL-C dynamics and changes in key clinical and echocardiographic parameters, we believe the influence of these potential confounding factors on the results is negligible.

## 5 Conclusions

In this prospective, randomized study, statin therapy with atorvastatin or rosuvastatin (primarily at high doses) was associated with improved functional capacity in ambulatory patients with chronic, stable HFpEF. These improvements appeared to result from beneficial effects on LV filling pressures and key cardiac reserves. Atorvastatin therapy likely had a greater impact on clinical, hemodynamic, and biomarker variables than rosuvastatin therapy. Our results suggest that patients with HFpEF may benefit from statin therapy. Nevertheless, given the limitations mentioned above, these results should be considered hypothesis-generating and primarily supportive of a potential role for statin therapy in HFpEF. Further research on statins, including head-to-head trials comparing different statin types in this population, is warranted.

## Data Availability

The raw data supporting the conclusions of this article will be made available by the authors, without undue reservation.
