# Supplementary material for: Effects of statins on functional capacity and cardiac remodeling in patients with heart failure and preserved ejection fraction: a randomized open-label pilot trial
Source: Front Cardiovasc Med. 2026 Jul 13;13:1870027. doi: 10.3389/fcvm.2026.1870027 (PMC13402443; doi:10.3389/fcvm.2026.1870027)
Supplement: Supplementary file 1 [file Table1.docx]

Supplementary Material

Supplementary Table. Correlations of LDL-C level with key clinical and echocardiographic parameters.

| Independent parameter | Dependent parameter | Atorvastatin (n=30) | | Rosuvastatin (n=29) | |
| --- | --- | --- | --- | --- | --- |
|  |  | r | P Value | r | P Value |
| Change in LDL-C from baseline | Change in 6MWTD  Change in bicycle exercise duration  Change in NT-proBNP  Change in LA volume index  Change in mitral E/e′ ratio  Change in PASP | 0.10  0.06  –0.07  0.12  –0.17  –0.22 | 0.78  0.76  0.73  0.52  0.42  0.25 | 0.28  0.18  –0.31  0.16  –0.31  –0.23 | 0.10  0.34  0.11  0.41  0.079  0.22 |

Е, early inflow velocity; e′, averaged annulus relaxation velocity; LAVI, left atrial volume index; LDL-C, low-density lipoprotein cholesterol; LV, left ventricular; NT-proBNP, N-terminal pro–brain natriuretic peptide; PASP, pulmonary artery systolic pressure; r, correlation coefficient; 6MWTD, 6-minute walk test distance
